# All for one or some for all? Evaluating informative hypotheses using multiple *N* = 1 studies

**DOI:** 10.3758/s13428-017-0992-5

**Published:** 2017-12-15

**Authors:** Fayette Klaassen, Claire M. Zedelius, Harm Veling, Henk Aarts, Herbert Hoijtink

**Affiliations:** 1 Department of Methodology and Statistics, Utrecht University, PO Box 80140, 3508 TC Utrecht, The Netherlands; 2 Department of Psychology, University of California, Santa Barbara, CA USA; 3 Behavioural Science Institute, Radboud University, Nijmegen, The Netherlands; 4 Department of Psychology, Utrecht University, Utrecht, The Netherlands; 5 Cito Institute for Educational Testing, Arnhem, The Netherlands

**Keywords:** Bayes factor, Informative hypotheses, *N* = 1 studies, Within-subject experiment

## Abstract

Analyses are mostly executed at the population level, whereas in many applications the interest is on the individual level instead of the population level. In this paper, multiple *N* = 1 experiments are considered, where participants perform multiple trials with a dichotomous outcome in various conditions. Expectations with respect to the performance of participants can be translated into so-called informative hypotheses. These hypotheses can be evaluated for each participant separately using Bayes factors. A Bayes factor expresses the relative evidence for two hypotheses based on the data of one individual. This paper proposes to “average” these individual Bayes factors in the gP-BF, the average relative evidence. The gP-BF can be used to determine whether one hypothesis is preferred over another for all individuals under investigation. This measure provides insight into whether the relative preference of a hypothesis from a pre-defined set is homogeneous over individuals. Two additional measures are proposed to support the interpretation of the gP-BF: the evidence rate (ER), the proportion of individual Bayes factors that support the same hypothesis as the gP-BF, and the stability rate (SR), the proportion of individual Bayes factors that express a stronger support than the gP-BF. These three statistics can be used to determine the relative support in the data for the informative hypotheses entertained. Software is available that can be used to execute the approach proposed in this paper and to determine the sensitivity of the outcomes with respect to the number of participants and within condition replications.

## Introduction

There is increasing attention for individual-centered analyses (e.g., Molenaar, 2004; Hamaker, 2012). For example, in personalized medicine, it is not relevant to find if a treatment works *on average* in a group of individuals but rather whether it works for any individual (Woodcock, 2007). This paper is concerned with individual-centered analyses in the form of multiple *N* = 1 studies. A core feature of this paper is that multiple hypotheses are formulated for each person. These hypotheses are first evaluated at the individual level and subsequently conclusions are formed at the group level. Specifically, this will be done in the context of a within-subject experiment (see Kluytmans et al., 2014, for a pilot study into using informative hypothesis in the context of multiple *N* = 1 studies). In a within-subject experiment each person *i* = 1,...,*P* is exposed to the same set of experimental conditions *j* = 1,…,*J*. By conducting *R* replications with a dichotomous outcome (0 = failure, 1 = success) in condition *j* the number of successes ${x_{j}^{i}}$ of person *i* can be obtained. This can be modeled using a binomial model with *R* trials and unknown success probability ${{\pi }_{j}^{i}}$.

This paper proposes a Bayesian method that evaluates informative hypotheses (Hoijtink, 2012) for multiple within-subject *N* = 1 studies. Researchers can formulate informative hypotheses based on (competing) theories or expectations. This can be achieved by using the relations ‘>’ and ‘<’ to impose constraints on the parameters $\boldsymbol {\pi }^{i} = [{{\pi }_{1}^{i}},\ldots ,{{\pi }_{J}^{i}}]$. E.g. ‘${\pi _{1}^{i}} > {\pi _{2}^{i}}$’ states that ${{\pi }_{1}^{i}}$ is larger than ${{\pi }_{2}^{i}}$ and reversely, ‘${{\pi }_{1}^{i}} < {\pi _{2}^{i}}$’ states that ${{\pi }_{1}^{i}}$ is smaller then ${{\pi }_{2}^{i}}$. When a comma is used to separate two parameters, such as ‘${\pi _{1}^{i}}, {\pi _{2}^{i}}$’, no constraint is imposed between these parameters. For each person, multiple informative hypotheses can be evaluated by means of Bayes factors (Kass & Raftery, 1995). Using the Bayes factor, it can be determined for each person which hypothesis is most supported by the data. Here, our method departs from traditional analyses. Rather than evaluating hypotheses at the group level, the hypotheses are evaluated for each person separately. In social psychology, for example, it is often hoped or thought that if a hypothesis holds at the group level, this also applies to all individuals (see for example, Moreland & Zajonc, 1982; Klimecki, Mayer, Jusyte, Scheeff, & Schönenberg, 2016). Hamaker (2012) describes the importance of individual analyses using an example: Cross-sectionally, the number of words typed per minute and the percentage of typos might be negatively correlated. That is, people that type fast tend to be good at typing and thus make fewer mistakes than people that type slow. However, at the individual level, a positive correlation exists between these variables, i.e., if a fast typer goes faster than his normal typing speed, the number of mistakes will increase (Hamaker, 2012). Similarly, if multiple persons aim to score a penalty several times, we might find that the average success probability is smaller than 0.5, however this does not imply that each individual has a penalty scoring probability smaller than 0.5. Differently from Hamaker (2012) and Molenaar (2004), our approach does not stop at a single *N* = 1 study. Rather, when individual analyses have been executed, it is interesting to see if all individuals support the same hypothesis. Thus, when multiple hypotheses are evaluated for *P* individuals, two types of conclusions can be drawn. First, by executing multiple *N* = 1 studies, it can be determined for each person if any hypothesis can be selected as the best, and if so, which hypothesis this is. Second, it can be determined if the sample comes from a population that is homogeneous with respect to the support of the specified hypotheses, and if so, which hypothesis is supported most.

This paper is structured as follows: First, the difference between analyses at the group level and multiple *N* = 1 analyses is elaborated upon by means of an example that will be used throughout the paper. Second, it will be described how informative hypotheses can be evaluated for one *N* = 1 study. Third, it will be explained how multiple *N* = 1 studies can be used to evaluate each hypothesis and detect if any can be selected as the best hypothesis for all individuals. The appropriate number of replications and the number of participants can be determined using a sensitivity analysis. The paper is concluded with a short discussion.

## P-population and WP-population

An example of a within-subject experiment is Zedelius, Veling, and Aarts (2011). These researchers investigated the effect of interfering information and reward on memory. In each trial, participants were shown five words on a screen and asked to remember these for a brief period of time. During this time, interfering information was presented on the screen. Afterwards, they were asked to recall the five words verbally in order to obtain a reward. Three factors with two levels each were manipulated over the trials: Before each trial started, participants were shown a *high* (hr) or a *low* (lr) reward on the screen they would receive upon completing the task correctly. This reward could be displayed *subliminally* (sub), that is, very briefly (17 ms) or *supraliminally* (sup), that is for a longer duration of 300 ms. Finally, the visual stimulus interfering with the memory task was either a sequence of letters, *low interference* (li), or eight words that were different from the five memorized *high interference* (hi). Combining these factors results in eight conditions, for example *hr-sub-hi* and *lr-sup-li*. Seven trials were conducted in each condition, resulting in a total of 56 trials per participant. After each trial, the participant was given a score of 1 if all five words were recalled and 0 if not.

Zedelius et al., (2011) specified expectations regarding the ordering of success probabilities that can be translated in many different hypotheses. One example of an informative hypothesis based on the expectations of Zedelius et al., (2011) is

1 $$\begin{array}{@{}rcl@{}} H_{1}:\textit{hr-sup-li} \!&>&\! \textit{hr-sup-hi} \!>\! \textit{hr-sub-li} \!>\! \textit{hr-sub-hi}\\ &>&\! \textit{lr-sup-li} \!>\! \textit{lr-sup-hi} \!>\! \textit{lr-sub-li} \!>\! \textit{lr-sub-hi},\\ \end{array} $$

where *hr-sup-li* is *π*_hr-sup-li_, the success probability in condition hr-sup-li. For simplifications in the remainder of this paper, *π* is omitted in the notation of all examples using the conditions from Zedelius et al., (2011). Alternatively, for each person *i* the hypothesis could be formulated as:

2 $$\begin{array}{@{}rcl@{}} {H_{1}^{i}}: \textit{hr-sup-li}^{i} \!&>&\! \textit{hr-sup-hi}^{i} \!>\! \textit{hr-sub-li}^{i} \!>\! \textit{hr-sub-hi}^{i}\\ &>&\! \textit{lr-sup-li}^{i} \!>\! \textit{lr-sup-hi}^{i} \!>\! \textit{lr-sub-li}^{i} \!>\! \textit{lr-sub-hi}^{i},\\ \end{array} $$

where *hr-sup-li* ^*i*^ is the success probability in condition hr-sup-li of person *i*.

To illustrate the difference between Eqs. 1 and 2 let us consider a *population of persons* (P-population from here on) and a *within-person population* (WP-population from hereon). Each individual in the P-population has their own success probabilities ***π*** ^*i*^. The averages of these individual probabilities are the P-population probabilities ***π*** = [*π*_1_,...,*π*_*J*_], where $\pi _{j} = \frac {1}{P} {\sum }_{i = 1}^{P}{\pi _{j}^{i}}$. Equation 1 is a hypothesis regarding the ordering of these P-population probabilities. Equation 2 is a hypothesis regarding the ordering of the WP-population probabilities for person *i*. Evaluating this hypothesis for person *i* is an example of an *N* = 1 study.

Many statistical methods are suited to draw conclusions at the P-population level. However, if a hypothesis is true at the P-population level, there is no guarantee that it holds for all WP-populations (Hamaker, 2012). Thus, a conclusion at the P-population level does not necessarily apply to each individual. Rather than ***π***, this paper concerns the individual ***π*** ^*i*^. If multiple hypotheses are formulated for each person *i*, it can be determined for each person which hypothesis is most supported. Furthermore, it can be assessed whether the sample of *P* persons comes from a population that is homogeneous with respect to the informative hypotheses under consideration.

## N = 1: how to analyze the data of one person

This section describes how the data of one person can be analyzed. First, the general form of hypotheses considered for every person are introduced. Subsequently, the statistical model used to model the *N* = 1 data is introduced. Finally, the Bayes factor is introduced and elaborated upon.

### Hypotheses

Researchers can formulate informative hypotheses regarding ***π*** ^*i*^. The general form of the informative hypotheses used in this paper is:

3 $$ {{H}_{m}^{i}}: {\boldmath{R}_{m}} \boldsymbol{\pi}^{i} > 0,  $$

where *m*,*m* ^′^ = 1,...,*M*(*m*≠*m* ^′^) is the label of a hypothesis, *M* is the number of hypotheses considered and *m* ^′^ is another hypothesis than *m*, $\boldsymbol {\pi }^{i} = [{\pi ^{i}_{1}}, ... {\pi ^{i}_{J}}]$ and *R*_*m*_ is the constraint matrix with *J* columns and *K* rows, where *K* is the number of constraints in a hypothesis. The constraint matrix can be used to impose constraints on (sets of) parameters. An example of a constraint matrix *R* for *J* = 4 is:

4 $$ {\boldmath{R}_{1}} = \left[\begin{array}{cccc} 1 & -1 & 0 & 0 \\ 0 & 1 & -1 & 0 \\ 0 & 0 & 1 & -1 \end{array}\right], $$

which renders

5 $$ {{H}_{1}^{i}}: {{\pi}_{1}^{i}} > {{\pi}_{2}^{i}} > {{\pi}_{3}^{i}} > {{\pi}_{4}^{i}},  $$

which specifies that the success probabilities ***π*** ^*i*^ are ordered from large to small. Note that the first row of *R*_1_ specifies that $1 \cdot {{\pi }_{1}^{i}} - 1 {\cdot {\pi }_{2}^{i}} + 0 \cdot {{\pi }_{3}^{i}} + 0 \cdot {{\pi }_{4}^{i}} > 0$, that is, ${{\pi }_{1}^{i}}>{{\pi }_{2}^{i}}$. The constraint matrix

6 $$ {\boldmath{R}}_{2} = \left[\begin{array}{cccc} .5 & .5 & -.5 & -.5 \end{array}\right], $$

renders the informative hypothesis

7 $$ {{H}_{2}^{i}}: \frac{{{\pi}_{1}^{i}} + {{\pi}_{2}^{i}}}{2} > \frac{{{\pi}_{3}^{i}} + {{\pi}_{4}^{i}}}{2},  $$

which states that the average of the first two success probabilities is larger than the average of the last two. Hypotheses constructed using Eq. 3 are a translation of the expectations researchers have with respect to the outcomes of their experiment into restrictions on the elements of ***π*** ^*i*^.

Another hypothesis that is considered in this paper is the complement of an informative hypothesis: 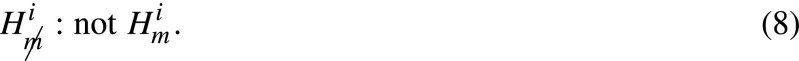


The complement states that ${{H}_{m}^{i}}$ is not true in the WP-population. Stated otherwise, the reverse of the researchers’ expectation is true. Finally, ${H}_{u}^{i}$ denotes the unconstrained hypothesis:

9 $$ {{H}_{u}^{i}}: {{\pi}_{1}^{i}}, {{\pi}_{2}^{i}}, \ldots, {\pi}_{J-1}^{i}, {{\pi}_{J}^{i}}, $$

where each parameter is ‘free’. An informative hypothesis ${{H}_{m}^{i}}$ constrains the parameter space such that only particular combinations of parameters are allowed, comprises that part of the parameter space that is not included in ${{H}_{m}^{i}}$ and the conjunction of ${{H}_{m}^{i}}$ and is ${{H}_{u}^{i}}$. The difference in use of ${H}_{u}^{i}$ and will be elaborated further in the section on Bayes factors.

**Fig. 1:**
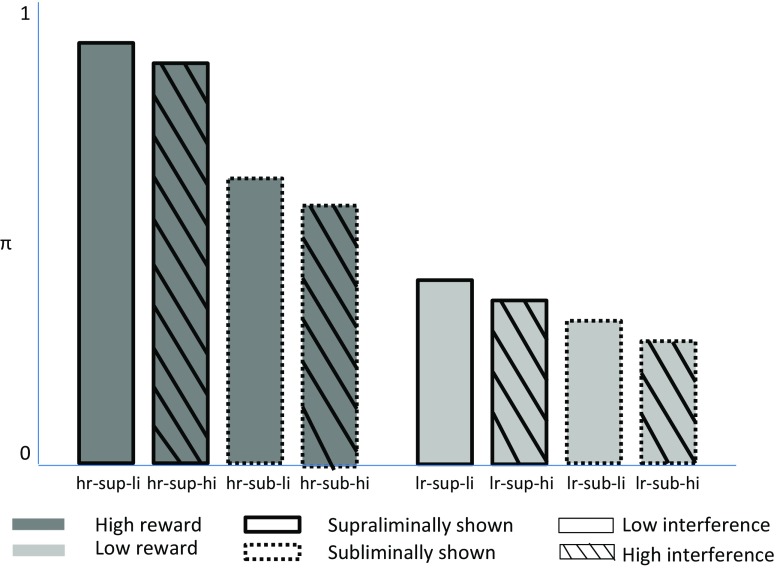
Graphical representation of all hypotheses by Zedelius et al., (2011)

Zedelius et al., (2011) formulated several expectations concerning the ordering of success probabilities over the experimental conditions. The main expectation was that high-reward trials would have a higher success probability than low-reward trials. This main effect and the expectations regarding the other conditions (interference level and visibility duration) can be translated in various informative hypotheses (Kluytmans et al., 2014). A first translation of the expectations is

10 $$\begin{array}{@{}rcl@{}} {H_{1}^{i}}: \textit{hr-sup-li}^{i} \!&>&\! \textit{hr-sup-hi}^{i} \!>\! \textit{hr-sub-li}^{i} \!>\! \textit{hr-sub-hi}^{i}\\ &>&\!\textit{lr-sup-li}^{i} \!>\! \textit{lr-sup-hi}^{i} \!>\! \textit{lr-sub-li}^{i} \!>\! \textit{lr-sub-hi}^{i},\\ \end{array} $$

which states that for any person *i* the success probabilities are ordered from high to low. To give some intuition for this hypothesis, Fig. 1 shows eight bars that represent the experimental conditions, and its height indicates the success probability in that condition, and the ordering of probabilities adheres to ${{H}_{1}^{i}}$. Substantively, this hypothesis specifies that all conditions with a high reward have a higher success probability than those with a low reward, which in Fig. 1 can be verified since all dark gray bars are higher than any light gray bar. Furthermore, ${{H}_{1}^{i}}$ specifies that within this main reward value effect, that is, looking only at high-reward success conditions or only at low-reward conditions, a supraliminally shown rewards (solid border) results in a higher success probability than a subliminally shown reward (dotted border). Finally, within the visibility duration effect, that is, looking only at conditions with the same reward and same visibility duration, low interference (no pattern) results in a higher success probability than high interference (diagonally striped pattern). Alternatively, two less-specific hypotheses can be formulated that include the main effect of reward and only one of the remaining main effects:

11 $$ {H_{2}^{i}}: \textit{hr-li}^{i} > \textit{hr-hi}^{i} > \textit{lr-li}^{i} > \textit{lr-hi}^{i},  $$

and

12 $$ {H_{3}^{i}}: \textit{hr-sup}^{i} > \textit{hr-sub}^{i} > \textit{lr-sup}^{i} > \textit{lr-sub}^{i},  $$

where *hr-li* ^*i*^ indicates the average success probability of the *hr-sup-li* ^*i*^ and *hr-sub-li* ^*i*^ conditions. In Fig. 1, both ${{H}_{2}^{i}}$ and ${{H}_{3}^{i}}$ are true. Different from ${{H}_{1}^{i}}$, these hypotheses do not state that *any* high-reward condition has a higher success probability than *any* low-reward condition, but rather that averaged over both interference level and visibility duration high-reward conditions have a higher success probability than low-reward conditions. Additionally, ${{H}_{2}^{i}}$ further specifies that averaged over visibility duration, the success probability is always higher in high-reward conditions compared to low-reward conditions. Within this main effect of reward value, the success probability is higher for low interference than for high interference. Analogously, ${{H}_{3}^{i}}$ states that averaged over interference level, the success probability is always larger in high- compared to low-reward conditions. Within this pattern, the success probability is larger for supraliminally compared to subliminally shown rewards.

A fourth hypothesis relates to the interaction effect between reward type and visibility duration:

13 $$ {H_{4}^{i}}: \textit{hr-sup}^{i} - \textit{lr-sup}^{i} > \textit{hr-sub}^{i} - \textit{lr-sub}^{i},  $$

which states that the benefit of high reward over low reward is larger when the reward is shown supraliminally compared to when the reward is shown subliminally. This, too, is presented in Fig. 1, since the difference between *hr-sup* (average of the dark-gray, solid border bars) and *lr-sup* (average of the light-gray, solid border bars) is larger than the difference between *hr-sub* (average of the dark-gray, dashed border bars) and *lr-sub* (average of the light-gray, dashed border bars). Note that, other than ${{H}_{2}^{i}}$ and ${{H}_{3}^{i}}$, ${{H}_{1}^{i}}$ is not a special case of ${{H}_{4}^{i}}$. These hypotheses can both be true, as is presented in the figure, but knowing that ${H_{1}^{i}}$ is true gives no information about ${H_{4}^{i}}$.

Together, ${{H}_{1}^{i}}$, ${{H}_{2}^{i}}$, ${{H}_{3}^{i}}$ and ${{H}_{4}^{i}}$ form a set of competing informative hypotheses that can be evaluated for each person.

### Density, prior, posterior

To evaluate hypotheses using a Bayes factor, the density of the data, prior and posterior distribution are needed. For the type of data used in this paper, that is, the number of successes **x** ^*i*^ = [*x*1*i*,…,*x* *J* *i*] observed for person *i* in *R* replications in each condition *j* the density of the data is

14 $$ f(\mathbf{x}^{i}\mid\boldsymbol{\pi}^{i})=\prod\limits_{j = 1}^{J} \left( \begin{array}{c}R\\{x_{j}^{i}} \end{array}\right) ({\pi_{j}^{i}})^{{x_{j}^{i}}}(1-{\pi_{j}^{i}})^{R-{x_{j}^{i}}},  $$

that is, in each condition *j* the response ${x_{j}^{i}}$ is modeled by a binomial distribution. The prior distribution $h(\boldsymbol {\pi }^{i}|{H_{u}^{i}})$ for person *i* is a product over Beta distributions

15 $$ h(\boldsymbol{\pi}^{i}\mid {H_{u}^{i}})= \prod\limits_{j = 1}^{J}\frac{\Gamma(\alpha_{0} + \beta_{0})}{\Gamma(\alpha_{0}) {\Gamma}(\beta_{0})}({\pi_{j}^{i}})^{\alpha_{0}-1}(1-{\pi_{j}^{i}})^{\beta_{0}-1},  $$

where *α*_0_ = *β*_0_ = 1, such that $h(\boldsymbol {\pi }^{i}\mid {H_{u}^{i}})= 1$, that is, a uniform distribution. As will be elaborated upon in the next section, only $h(\boldsymbol {\pi }^{i}\mid {H_{u}^{i}})$ is needed for the computation of the Bayes factors involving ${H_{m}^{i}}$, $H_{m^{\prime }}^{i}$ and $H_{u}^{i}$ (Klugkist, Laudy, & Hoijtink, 2005). The interpretation of *α*_0_ and *β*_0_ is the prior number of successes and failures plus one. In other words, using *α*_0_ = *β*_0_ = 1 implies that the prior distribution is uninformative. Consequently, the posterior distribution based on this prior is completely determined by the data. Furthermore, by using *α*_0_ = *β*_0_ = 1 for each ***π*** ^*i*^ the prior distribution is unbiased with respect to informative hypotheses that belong to an equivalent set (Hoijtink, 2012, p. 205). As will be elaborated in the next section, unbiased prior distributions are required to obtain Bayes factors that are unbiased with respect to the informative hypotheses under consideration.

The unconstrained posterior distribution is proportional to the product of the prior distribution and the density of the data:

16 $$\begin{array}{@{}rcl@{}} g(\boldsymbol{\pi}^{i}\mid \mathbf{x}^{i}, {H_{u}^{i}}) &\propto& f(\mathbf{x}^{i}\mid \boldsymbol{\pi}^{i})\cdot h(\boldsymbol{\pi}^{i}\mid {H_{u}^{i}})\\ &\propto& \prod\limits_{j = 1}^{J} \frac{\Gamma(\alpha_{1} + \beta_{1})}{\Gamma(\alpha_{1}){\Gamma}(\beta_{1})} ({\pi_{j}^{i}})^{\alpha_{1}-1} (1-{\pi_{j}^{i}})^{\beta_{1}-1},\\ \end{array} $$

where $\alpha _{1} = {{x}_{j}^{i}} + \alpha _{0} = {{x}_{j}^{i}}+ 1$ and $\beta _{1}=(R-{{x}_{j}^{i}}) + \beta _{0}=(R-{{x}_{j}^{i}}) + 1$. As can be seen in Eq. 15, the posterior distribution is indeed only dependent on the data.

### Bayes factor

We will use the Bayes factor to evaluate informative hypotheses. A Bayes factor (BF) is commonly represented as the ratio of the marginal likelihoods of two hypotheses (Kass & Raftery, 1995). Klugkist et al., (2005) and Hoijtink (2012, p. 51–52, 57–59) show that for inequality constrained hypotheses of the form presented in Eq. 3 the ratio of marginal likelihoods expressing support for ${H_{m}^{i}}$ relative to $H_{u}^{i}$ can be rewritten as

17 $$ BF_{mu}^{i} = \frac{{f_{m}^{i}}}{{c_{m}^{i}}}.  $$

The Bayes factor balances the relative fit and complexity of two hypotheses. Fit and complexity are called relative because they are relative with respect to the unconstrained hypothesis. In the remainder of this text, referrals to fit and complexity should be read as *relative* fit and complexity. The complexity ${{c}_{m}^{i}}$ is the proportion of the unconstrained prior distribution for ${{H}_{u}^{i}}$ in agreement with ${{H}_{m}^{i}}$

18 $$ {{c}_{m}^{i}} = {\int}_{\boldsymbol{\pi}^{i}\in {H_{m}^{i}}} h(\boldsymbol{\pi}^{i}\mid {H_{u}^{i}})\delta\boldsymbol{\pi}^{i}.  $$

Using Eq. 14 with *α*_0_ = *β*_0_ = 1 for each ***π*** ^*i*^ it is ensured that the prior distribution is unbiased with respect to hypotheses that belong to an equivalent set. Consider for example, *H*_1_ : *π*_1_ > *π*_2_ > *π*_3_ > *π*_4_ and *H*_2_ : *π*_1_ > *π*_2_ > *π*_4_ > *π*_3_. These hypotheses, and the other 22 possible ordering of ***π*** ^*i*^, are equally complex and should thus have the same complexity. Using Eq. 14, this complexity is computed as $\frac {1}{24}$ for each of the set of 24 equivalent hypotheses (Hoijtink, 2012, p. 60).

The fit ${{f}_{m}^{i}}$ is the proportion of the unconstrained posterior distribution in agreement with ${{H}_{m}^{i}}$:

19 $$ {f_{m}^{i}} ={\int}_{\boldsymbol{\pi}^{i}\in {H_{m}^{i}}} g(\boldsymbol{\pi}^{i}\mid \mathbf{x}^{i}, {H_{u}^{i}})\delta \boldsymbol{\pi}^{i}. $$

The Appendix describes how stable estimates of the complexity and fit can be computed using MCMC samples from the prior and posterior distribution, respectively.

Since Eq. 16 is a ratio of two marginal likelihoods (one for ${H_{m}^{i}}$ and one for ${H_{u}^{i}}$) it follows that

20 $$ BF_{mm^{\prime}}^{i} = \frac{BF_{mu}^{i}}{BF_{m^{\prime}u}^{i}}=\frac{{f_{m}^{i}}/{c_{m}^{i}}}{f_{m^{\prime}}^{i}/c_{m^{\prime}}^{i}},  $$

and that 
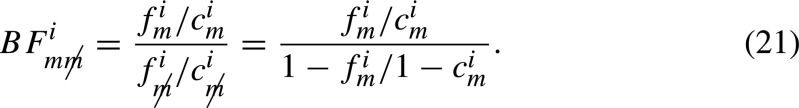


Three hypothetical *N* = 1 datasets with *J* = 4 and *R* = 7 are presented in Table 1. Three possible informative hypotheses regarding these data are ${{H}_{1}^{i}}$ from Eq. 5, and ${H_{2}^{i}} $ from Eq. 7. The table presents the complexity, fit and Bayes factors of these hypotheses. As can be seen in the table, the complexity of ${H_{1}^{i}}$ is .04 = 1/24 and ${c_{2}^{i}} = .5$. The table illustrates that complexity depends on the hypotheses but not on the data: for each of the three data examples the complexities are the same.

**Table 1:** Complexity, fit, and Bayes factors for three hypothetical *N* = 1 studies with ${{H}_{1}^{i}} = {{\pi }_{1}^{i}} > {{\pi }_{2}^{i}} > {{\pi }_{3}^{i}} > {{\pi }_{4}^{i}}$ and ${{H}_{2}^{i}} = \frac {{{\pi }_{1}^{i}} + {{\pi }_{2}^{i}}}{2} > \frac {{{\pi }_{3}^{i}} + {{\pi }_{4}^{i}}}{2}$

*i*	${x_{1}^{i}}$	${x_{2}^{i}}$	${x_{3}^{i}}$	${x_{4}^{i}}$	${c_{1}^{i}}$	${c_{2}^{i}}$	${f_{1}^{i}}$	${f_{2}^{i}}$	$BF_{1u}^{i}$	$BF_{2u}^{i}$		$BF_{12}^{i}$	
1	7	5	4	1	.04	.50	.56	.99	13.16	2.00	28.39	6.59	99
2	7	2	5	1	.04	.50	.06	.89	1.40	1.79	1.43	.78	8.09
3	3	4	6	1	.04	.50	.01	.51	.24	1.01	.23	.24	1.04

The first example (Person 1) in Table 1 contains data that are in agreement with ${H_{1}^{i}}$, and therefore also with ${H_{2}^{i}}$, since ${H_{1}^{i}}$ is a specific case of ${H_{2}^{i}}$. This is reflected by ${f_{1}^{1}} = .556$ and ${f_{2}^{1}} = .996$. Because ${H_{1}^{i}}$ is quite specific, it can easily conflict with the data. For example, based on ${x_{2}^{1}} = 5$ and ${x_{3}^{1}}= 4$, it is not very certain that ${\pi _{2}^{1}}>{\pi _{3}^{1}}$. In contrast, ${H_{2}^{i}} $ is less specific, does not involve the constraint ${\pi _{2}^{1}} > {\pi _{3}^{1}}$, and therefore ${f_{2}^{1}}$ is larger than ${f_{1}^{1}}$. Bayes factors balance complexity and fit of the hypotheses, resulting in $BF_{1u}^{1} = 13.16$, $BF_{2u}^{1}= 2.00$, $BF_{12}^{1} = 6.59$ and . Interpreting the size of Bayes factors is a matter that needs some discussion. Firstly, it is important to distinguish the different interpretations of $BF_{mu}^{i}$, $BF_{mm^{\prime }}^{i}$ and . In itself, $BF_{mu}^{i}$ represents the relative change in the support for ${H_{m}^{i}}$ and $H_{u}^{i}$ caused by the data. For example, in Table 1 we find that the belief for ${H_{1}^{1}}$ has increased 13 times and the belief for ${H_{2}^{1}}$ has increased 2 times. This shows that, although with varying degrees, both hypotheses are supported by the data. If we compute $BF_{mm^{\prime }}^{i}$ we can quantify the relative change in support for ${H_{m}^{i}}$ and $H_{m^{\prime }}^{i}$ caused by the data. For example, $BF_{12}^{1} = 6.6$, indicating that the relative support for ${H_{1}^{1}}$ compared to ${H_{2}^{1}}$ has increased by a factor 6.6. However, $BF_{12}^{i}$ is only a relative measure of support, that is, the best of the hypotheses involved may still be an inadequate representation of the within person population that generated the data. Note that $BF_{mu}^{i}$ and are always both larger or smaller than 1. However, by definition $BF_{mu}^{i}$ ranges from 0 to $\frac {1}{c_{m}^{i}}$ and ranges from 0 to infinity. Therefore, we prefer to interpret the latter to determine if the best of a set of hypotheses is also a good hypothesis. By computing , we can determine whether the best hypothesis, in this case ${H_{m}^{i}}$, is also a good hypothesis, because we get an answer to the question “is or isn’t ${H_{m}^{i}}$ supported by the data?”. In Table 1, indicates that the data caused an increase in believe for ${H_{m}^{i}}$ compared to , which implies that it is a good hypothesis. Note that this does not rule out the possibility of other, perhaps better, good hypotheses.

A second issue is the interpretation of the strength of Bayes factors. Although some guidelines have been provided (e.g. Kass & Raftery, 1995, interpret 3 as the demarcation for the size of *B* *F*_*ab*_, providing marginal and positive evidence in favor of *H*_*a*_), we choose not to follow them. In the spirit of a famous quote from Rosnow and Rosenthal (1989), “surely God loves a BF of 2.9 just as much as a BF of 3.1”, we want to stay away from cut-off values in order not to provide unnecessary incentives for publication bias and sloppy science (Konijn, Van de Schoot, Winter, & Ferguson, 2015). In our opinion, claiming that a Bayes factor of 1.5 is not very strong evidence and that a Bayes factor of 100 is strong evidence will not result in much debate. It is somewhere between those values that scientists may disagree about the strength. In this paper, we used the following strategy to decide when a hypothesis can be considered best for a person: a hypothesis *m* is considered the best of a set of *M* hypotheses if the evidence for *H*_*m*_ is at least *M* − 1 times (with a minimum value of 2) stronger than for any other hypothesis *m* ^′^. This requirement ensures that the posterior probability for the best hypothesis is at least .5 if all hypotheses are equally likely a priori. For example, if two hypotheses are considered, one should be at least two times more preferred than the other, resulting in posterior probabilities of at least .66 versus .33. If three hypotheses are considered, the resulting posterior probabilities will be at least .50 versus .25 and .25, which corresponds to a twofold preference of one hypothesis over both alternatives. For four hypotheses the posterior probabilities should be at least .50 versus .16, .16 and .16, corresponding to relative support of at least 3 times more for the best hypothesis than for any other hypothesis. Note that, although these choices seem reasonable to us, other strategies can be thought of and justified.

For Person 2 in Table 1 ${H_{2}^{i}}$ has gained slightly more belief than ${H_{1}^{i}}$, since $BF_{12}^{2} = .78$ (*B* *F*212 = 1.28). Based on this Bayes factor, ${H_{2}^{i}} $ is not convincingly the better hypothesis of the two. It is important to note that Bayes factors for different persons do not necessarily express support in favor of one or the other hypothesis. It is very possible that Bayes factors for different persons are indecisive. Looking at and , ${H_{2}^{i}}$ seems quite a good hypothesis, whereas ${H_{1}^{i}}$ is not much more supported than its complement. Finally, Person 3 in Table 1 shows data that do not seem to be in line with either ${H_{1}^{i}}$ or ${H_{2}^{i}}$. According to $BF_{1u}^{3} =.24$, the support for ${H_{1}^{3}}$ relative to ${H_{u}^{3}}$ has decreased after observing the data. According to $BF_{2u}^{3} = 1.01$, the data do not cause a change in support for ${H_{2}^{3}}$ relative to the unconstrained hypothesis. When we look at $BF_{12}^{3} = .24$ (*B* *F*213 = 4.17), we find that ${H_{2}^{i}} $ is a somewhat better hypothesis than ${H_{1}^{i}}$. However, , indicating that although ${H_{2}^{i}} $ is better than ${H_{1}^{i}}$, it is not a very good hypothesis. The examples in Table 1 show the variety in conclusions that can be obtained. There may or may not be a best hypothesis, and the best hypothesis may or may not be a good hypothesis.

### Illustration

For Zedelius et al., (2011), the main goal was to select the best hypothesis from ${H_{1}^{i}}$, ${H_{2}^{i}}$, ${H_{3}^{i}} $ and ${H_{4}^{i}}$ presented in Eqs. 9, 10, 11 and 12. The Bayes factors presented in the first four columns of Table 2 can be used to select the best hypothesis for each person. If a best hypothesis is selected, it is also of interest to determine whether this hypothesis is a good hypothesis. The last four columns of Table 2 can be used to determine whether the best hypothesis is also ‘good’.

**Table 2:** Individual Bayes factors for the Zedelius data where $H_{1}^{i}, H_{2}^{i}, H_{3}^{i}$ and $H_{4}^{i}$ (Eqs. 9–12) are evaluated against ${H}_{u}^{i}$ and their complement

*i*	$BF_{1u}^{i}$	$BF_{2u}^{i}$	$BF_{3u}^{i}$	*B* *F*4*u* *i*				
1	0.59	0.93	1.98	0.26	0.59	0.93	2.06	0.15
2	3.33	1.49	4.67	0.45	3.33	1.52	5.54	0.29
3	1.02	1.31	1.63	1.41	1.02	1.33	1.68	2.37
4	0.03	0.10	0.58	1.22	0.03	0.10	0.57	1.55
5	3.79	2.39	4.92	1.02	3.79	2.55	5.91	1.04
6	543.90	17.95	13.74	1.43	551.21	68.72	30.30	2.51
7	1.44	3.45	2.88	1.23	1.44	3.87	3.14	1.58
8	< 0.01	0.16	0.02	0.19	< 0.01	0.15	0.02	0.10
9	3.06	6.16	3.25	1.94	3.06	7.95	3.59	30.74
10	2.60	3.41	2.75	0.99	2.60	3.81	2.97	0.97
11	0.05	0.24	0.55	1.21	0.05	0.23	0.54	1.53
12	1.29	1.70	1.55	0.44	1.29	1.76	1.58	0.28
13	0.30	3.50	2.66	0.79	0.30	3.93	2.86	0.65
14	0.55	6.53	0.56	0.78	0.55	8.61	0.55	0.64
15	21.84	2.01	6.41	1.73	21.85	2.10	8.35	6.28
16	0.18	0.45	3.21	1.22	0.18	0.44	3.54	1.56
17	22.30	5.15	3.88	1.91	22.31	6.28	4.42	20.64
18	0.32	1.37	0.55	0.62	0.32	1.39	0.54	0.45
19	< 0.01	< 0.01	0.03	1.96	< 0.01	< 0.01	0.03	40.41
20	< 0.01	< 0.01	0.01	0.79	< 0.01	< 0.01	0.01	0.65
21	0.09	0.41	0.40	1.43	0.09	0.40	0.39	2.50
22	15.78	5.59	4.82	1.58	15.78	6.98	5.77	3.68
23	20.92	4.39	7.62	1.60	20.93	5.15	10.64	3.92
24	0.15	1.16	0.32	1.01	0.15	1.17	0.31	1.02
25	7.21	3.16	3.26	0.76	7.21	3.49	3.61	0.61
26	0.06	0.13	0.38	0.58	0.06	0.13	0.37	0.41

For Person 1, ${H_{3}^{1}}$ is 1.98/.59 ≈ 3.36 times more supported than ${H_{1}^{1}}$, 1.98/.93 ≈ 2.13 times more supported than ${H_{2}^{1}}$ and 1.98/.26 ≈ 7.62 times more supported than ${H_{4}^{1}}$. Although ${H_{3}^{1}}$ is more supported than the other three hypotheses, a Bayes factor of 2.13 does not seem very convincing. Comparing the relative strength of the support for all informative hypotheses for Person 1 leaves us with the conclusion that no single best hypothesis could be detected. This implies that for Person 1, we would not be quite certain which hypothesis best describes the data Thus, we may conclude that for Person 1, it is difficult to select a best hypothesis.

For Person 8, none of the informative hypotheses is preferred over the unconstrained hypothesis. Thus, for each of the formulated hypotheses, our belief has decreased after obtaining the data. If we have to select a best hypothesis, however ${H_{2}^{8}}$ and ${H_{4}^{8}}$ are respectively .16/.03 ≈ 5.3 and .19/.03 ≈ 6.3 times more supported than ${H_{3}^{8}}$ and at least .16/.01 ≈ .19/.01 ≈ 17 times more supported than ${H_{1}^{8}}$. However, based on and we can conclude that although ${H_{2}^{8}}$ and ${H_{4}^{8}}$ are convincingly preferred over the other two hypotheses, neither is a good hypothesis for this person.

For Person 14, $H_{2}^{14}$ is 6.53/.55 ≈ 11.9 times more supported than $H_{1}^{14}$, 6.53/.56 ≈ 11.7 times more supported than $H_{3}^{14}$ and 6.53/.78 ≈ 8.4 times more supported than $H_{4}^{14}$. We find that , so besides the fact that $H_{3}^{14}$ is preferred over the other hypotheses it is a good hypothesis, too. Thus, we may conclude that for Person 14 we can find a best hypothesis that appears to be a good hypothesis, too.

For Person 20, $H_{4}^{20}$ is at least 79 times more supported than $H_{1}^{20}$, $H_{2}^{20}$ and $H_{3}^{20}$. Thus, $H_{4}^{20}$ is the best hypothesis from the set. However, because we can conclude that even though $H_{4}^{20}$ was the best hypothesis, it is not a good description of the data.

These examples show that it differs per person whether a best hypothesis can be detected, which hypothesis this is, and how strong the evidence is relative to the other hypotheses. Based on Table 2, Zedelius et al., (2011) can conclude for each individual what the best hypothesis is, and whether it is a good hypothesis. We find that the sample contains persons for whom a best hypothesis can be detected, but this hypothesis is not a good hypothesis (Persons 20 and 21). Additionally, there are individuals for whom a best hypothesis can be detected and the best hypothesis is good (Persons 6, 14, 15, 16, 17, 19, 22, and 23). For the remaining individuals, no best hypothesis could be selected. Someone else evaluating these Bayes factors might come to slightly different conclusions, if they apply a different rule to decide what makes a hypothesis the best from a set.

The second goal of this paper was to determine whether the sample of individuals comes from a homogeneous population with respect to the support for the hypotheses of interest. The first impression gained from Table 2 is that this is not the case. However, this topic will be pursued in depth in the next section.

## A P-population of WP-populations

Looking at the Bayes factors in Table 2 is a rather ad hoc manner to answer the question whether the sample comes from a population that is homogeneous in its support for the hypotheses under consideration and which hypothesis is the best. By aggregating the individual Bayes factors we can try to evaluate in more detail to what extent individuals are homogeneous with respect to a hypothesis. If ${H_{m}^{i}}$ is evaluated for *P* independent persons the corresponding individual Bayes factors can be multiplied into a P-population Bayes factor (Stephan & Penny, 2007):

22 $$ \text{P-BF}_{mu} = \prod\limits_{i = 1}^{P} \text{BF}_{mu}^{i}, $$

which expresses the support for *H*_*m*_ relative to *H*_*u*_, where

23 $$ H_{m} = {H_{m}^{1}} \cup {\ldots} \cup {H_{m}^{P}}, $$

which states that ${{H}_{m}^{i}}$ holds for every person *i* = 1,…,*P*, and

24 $$ H_{u} = {H_{u}^{1}} \cup {\ldots} \cup {H_{u}^{P}}, $$

which is the union of ${H_{u}^{i}}$ for *i* = 1,…,*P*. In this section, using the Bayes factor, ${H_{m}^{i}}$ and *H*_*m*_ are compared with $H_{u}^{i}$ and *H*_*u*_, respectively. However, analogously, $H_{u}^{i}$ could be replaced by $H_{m^{\prime }}^{i}$ or rendering P-$BF_{mm^{\prime }}$ and , respectively. Note, that this is *not* the Bayes factor describing the relative evidence for Hm and Hm’ with regard to the P-population parameters ***π***. Individual data *could* be used to evaluate a Bayes factor with respect to the P-population ***π***, but our focus here is on the collection of individual WP-populations ***π*** ^*i*^. Another way to interpret this P-BF is in the context of *synthesis* of knowledge with respect to the individual evaluated hypotheses ${H_{m}^{i}}$. Thus, it is a measure of the extent to which a hypothesis holds for every individual, rather than on average.

Table 3 shows seven hypothetical sets of six individual Bayes factors comparing ${H_{m}^{i}}$ to $H_{u}^{i}$. The P-BF is presented for each set. For example, Set 1 results in a P-BF of 64, indicating that it is 64 times more likely that ${H_{m}^{i}}$ holds for all persons *i*, than that it does not hold for all persons. However, the table shows an undesirable property of P-BF, namely that it is a function of *P*. As can be seen, both in Set 1, 2 and 3, the P-BF is 64. Nevertheless, it is clear that all individual Bayes factors in Set 1 express stronger evidence than in Sets 2 and 3.

**Table 3:** Hypothetical individual Bayes factors (*P* = 6), gP-BF_*mu*_, ER_*mu*_ and SR_*mu*_ underlined entries indicate individual Bayes factors smaller than the gP-BF and bold entries indicate entries larger than the corresponding gP-BF

	Set 1	Set 2	Set 3	Set 4	Set 5	Set 6	Set 7
$BF^{1}_{mu}$	**9.00**	**3.20**	1.40	0.80	0.90	**6.40**	1.01
$BF^{2}_{mu}$	7.11	2.70	**2.70**	1.50	0.93	1.40	1.30
$BF^{3}_{mu}$	–	2.30	1.80	**2.50**	0.85	1.80	**2.50**
$BF^{4}_{mu}$	–	**3.22**	**2.10**	**4.33**	0.88	1.40	**3.10**
$BF^{5}_{mu}$	–	–	1.60	**3.10**	**6.30**	1.60	**2.60**
$BF^{6}_{mu}$	–	–	**2.80**	1.59	**16.23**	1.77	**2.42**
P-*B* *F*_*mu*_	64.00	64.00	64.00	64.00	64.00	64.00	64.00
gP-*B* *F*_*mu*_	8.00	2.83	2.00	2.00	2.00	2.00	2.00
*E* *R*_*mu*_	1	1	1	.83	.33	1	1
*S* *R*_*mu*_	.50	.50	.50	.50	.33	.17	.67

Stephan and Penny (2007) have suggested using the geometric mean of the product of individual Bayes factors to render a summary that is independent of *P*:

25 $$ \text{gP-BF}_{mu} = \sqrt[P]{\text{P-BF}_{mu}}, $$

which is a measure of the ‘average’ support in favor of *H*_*m*_ relative to *H*_*u*_ found in *P* persons. In other words, it can be interpreted as the Bayes factor that is expected for the *P* + 1^st^ individual sampled from the P-population.

As can be seen in Table 3, the gP-BF_*mu*_ does not depend on *P*. For example, in Set 1 the gP-BF is 8.00 and in the larger Sets 2 and 3, the average support for *H*_*m*_ is 2.83 and 2.00, respectively, while the P-*B* *F*_*mu*_ = 64 for each of these sets.

If multiple hypotheses are considered, gP-$BF_{mm^{\prime }}$ and can be derived similar as $BF_{mm^{\prime }}^{i}$ and . It is important to keep in mind that the gP-*B* *F*_*mu*_ is a summary measure and does not have the same properties as individual Bayes factors. Such a property is that $BF_{mu}^{i}$ and are always both smaller or larger than 1. For example, if $BF_{1u}^{1} = 0.2 $, then , and if $BF_{1u}^{2} = 1.8$ then . This is not true for gP-*B* *F*_*mu*_ and . To continue the example based on the Bayes factors for persons 1 and 2, gP-*B* *F*_1*u*_ = 0.6 and . For interpretation of the gP-*BF*, it is important to keep in mind that gP-*B* *F*_*mu*_ is a summary of all $BF_{mu}^{i}$, and thus cannot be translated into , which is a summary of all . Note that if a switch in direction occurs, both geometric Bayes factors are generally both close to 1, therefore not causing any very contradicting conclusions.

However, the gP-BF_*mu*_ has another issue. Table 3 shows that different sets of individual Bayes factors can lead to the same gP-BF_*mu*_. For example, in Sets 3, 4, and 5 the same gP-BF is obtained. Set 3 contains only Bayes factors that are close to the gP-BF = 2 and all support ${H_{m}^{i}}$. Set 4 seems similar in the strength of support in the individual Bayes factors, although there seems to be more variation than in Set 3, and we find one Bayes factor that does not support ${H_{m}^{i}}$. Finally, Set 5 contains four Bayes factors that express support for $H_{u}^{i}$ over ${H_{m}^{i}}$, while two Bayes factors express relatively strong support in favor of ${H_{m}^{i}}$ over ${H_{u}^{i}}$. The fact that the Bayes factors from Sets 3 and 4 come from populations that are more homogeneous in their preference for $H_{u}^{i}$ than Set 5 is not represented well by the gP-BF_*mu*_. Therefore, an additional measure, the evidence rate (ER_*mu*_), is introduced that describes the consistency in the preferred hypothesis in multiple individual Bayes factors:

26 $$ ER_{mu} = \begin{array}{ccc} \frac{1}{P} {\sum}_{i = 1}^{P}I_{BF_{mu}^{i} < 1} & \text{if} & \text{gP-BF}_{mu} < 1 \\ \frac{1}{P} {\sum}_{i = 1}^{P}I_{BF_{mu}^{i} > 1} & \text{if} & \text{gP-BF}_{mu} > 1 \end{array},  $$

where $I_{BF^{i}_{mu} > 1} = 1$ if $BF^{i}_{mu} > 1$ and 0 otherwise. Thus, the *E* *R*_*mu*_ is the proportion of individual $BF_{mu}^{i}$ that expresses support for ${H_{m}^{i}}$ or for $H_{u}^{i}$ if the gP-BF_*mu*_ expresses support for *H*_*m*_ or *H*_*u*_, respectively. For example, if gP-BF_*mu*_ > 1, an *E* *R*_*mu*_ of 1 indicates that all individual Bayes factors express support for ${H_{m}^{i}}$. An ER of .5, indicates that 50*%* of the individual Bayes factors expresses support for ${H_{m}^{i}}$, and 50% expresses support for ${H_{u}^{i}}$. An ER close to 1 indicates homogeneity among the individual Bayes factors. The lower the ER, the stronger the evidence that the ordering of the individual success probabilities are not homogeneous with respect to the hypotheses under consideration. Looking at Table 3, we find that in Set 3 all individual Bayes factors support ${H_{m}^{i}}$, this is reflected in an *E* *R*_*mu*_ = 1. In Set 4 most, but not all individual Bayes factors support ${H_{m}^{i}}$, resulting in *E* *R*_*mu*_ = .83. This implies that there is no perfect homogeneity among the individual Bayes factors. Finally, in Set 5, four of six individual Bayes factors support ${H_{u}^{i}}$, while gP-BF_*mu*_ supports ${H_{m}^{i}}$. The *E* *R*_*mu*_ of .33 indicates that Set 5 is not likely to come from a homogeneous population with respect to the hypotheses under consideration.

There is still one issue that needs to be resolved. Set 6 and 7 result in the same gP-BF_*mu*_ and ER_*mu*_ as Set 3, but are not similar in individual contributions. Set 6 contains an outlier that expresses strong evidence for ${H_{m}^{i}}$, whereas all other cases express only weak support for ${H_{m}^{i}}$. Without this outlier, the gP-BF_*mu*_ would be much lower. Set 7 contains two Bayes factors that express very little support for ${H_{m}^{i}}$, whereas the other four cases express stronger support for ${H_{m}^{i}}$. Without these two ‘weak’ cases, the gP-BF_*mu*_ would be somewhat higher. In contrast, Set 3 contains Bayes factors that are rather constant around gP-BF, removing any of these cases would not affect the gP-BF_*mu*_ too much. To describe presence and direction of skewness among individual Bayes factors with respect to the gP-BF_*mu*_, a final measure is introduced: the stability rate.

The stability rate (SR_*mu*_) is a measure of skewness among individual Bayes factors with respect to the gP-BF_*mu*_. It can be written as:

27 $$ SR_{mu} = \begin{array}{ccc} \frac{1}{P} {\sum}_{i = 1}^{P}I_{BF_{mu}^{i} < \text{gP-BF}_{mu}} & \text{if} & \text{gP-BF}_{mu} < 1 \\ \frac{1}{P} {\sum}_{i = 1}^{P}I_{BF_{mu}^{i} > \text{gP-BF}_{mu}} & \text{if} & \text{gP-BF}_{mu} > 1 \end{array},  $$

where $I_{BF^{i}_{mu} < \text {gP-BF}_{mu}} = 1$ if $BF^{i}_{mu} < \text {gP-BF}_{mu}$ and 0 otherwise. The SR_*mu*_ describes the proportion of individual Bayes factors that expresses support stronger than the gP-BF for the hypothesis preferred by gP-BF_*mu*_. In Sets 1, 2, 3, and 4 of Table 3 the gP-BF_*mu*_ prefers ${H_{m}^{i}}$ over ${H_{u}^{i}}$. Individual Bayes factors that express stronger support for ${H_{m}^{i}}$ than gP-BF are presented in bold in the table. For each of these sets, the SR_*mu*_ = .50, indicating that half of the individual Bayes factors expresses support for ${H_{m}^{i}}$ stronger than gP-BF. The other half expresses support either for $H_{u}^{i}$ or weaker support for ${H_{m}^{i}}$. An SR_*mu*_ close to .50 indicates that the individual Bayes factors are evenly distributed around gP-BF.

An SR_*mu*_ smaller than .50, as in Set 5 and 6, indicates that less than half of the individual Bayes factors express stronger support for ${H_{m}^{i}}$ than gP-BF. Consequently, the gP-BF_*mu*_ is relatively large because of a minority of individual Bayes factors that are relatively large. The gP-BF_*mu*_ is overestimated because of this minority. In Set 5 the gP-BF_*mu*_ supports ${H_{m}^{i}}$, while the majority of individual Bayes factors support ${H_{u}^{i}}$. The gP-BF_*mu*_ is no longer a representative ‘average’ support. Reversely, an SR_*mu*_ larger than .50 indicates that only relatively few individual Bayes factors express weaker support than gP-BF (see Set 7). Thus, for SR_*mu*_ > .50, the gP-BF_*mu*_ is relatively close to 1 because of a minority of individual Bayes factors that express support that is relatively weak. As an effect, the strength of support is underestimated.

Thus, the gP-BF_*mu*_ can be used to express the average support of the individual Bayes factors. In order to assess whether the individual Bayes factors come from a homogeneous population, the ER_*mu*_ can be used. A high evidence rate indicates high agreement in preferred hypothesis among individual Bayes factors, and thus more homogeneity. Finally, the SR_*mu*_ gives an indication of how the individual Bayes factors are distributed around the gP-BF_*mu*_. Note that the equations presented for the ER and SR describe those corresponding to gP-BF_*mu*_. If the interest is in gP-BF${~}_{mm^{\prime }}$ or , the ER and SR should be computed using the individual $BF_{mm^{\prime }}^{i}$s and . The individual Bayes factors are the relevant quantities in the ER and SR, and therefore these should be used.

### Illustration

Using the individual Bayes factors presented in Table 2 the gP-BF_*mu*_, ER_*mu*_ and SR_*mu*_ can be computed for the data of Zedelius et al., (2011). The first row of Table 4 gives the gP-BF_*mu*_ based on the individual Bayes factors from Table 2. The ER_*mu*_ and SR_*mu*_ are presented in the second and third row. Based on the gP-BF_*mu*_ we can conclude that *H*_3_ receives approximately 1.125/.510 ≈ 2.21 times more support than *H*_1_, and only about 1.125/.910 ≈ 1.125/.949 ≈ 1.2 times more support than *H*_2_ and *H*_4_. Thus, *H*_3_ is somewhat preferred over *H*_1_, but cannot be distinguished from *H*_2_ and *H*_4_. Furthermore, since , and , it can be concluded that none of the hypotheses is convincingly the best description for all individuals and none of the hypotheses are clearly a better description of all individuals than their complement is.

**Table 4:** The gP-BF, ER and SR for the data of Zedelius et al., (2011) for the evaluation of *H*1*i*,*H*2*i*,*H*3*i* and $H_{4}^{i}$ (Eqs. 9–12)

	*B* *F*_1 *u*_	*B* *F*_2 *u*_	*B* *F*_3 *u*_	*B* *F*_4 *u*_				
gP-BF	0.510	0.910	1.125	0.949	0.511	1.014	1.235	1.412
ER	0.500	0.346	0.615	0.423	0.500	0.654	0.615	0.577
SR	0.423	0.308	0.615	0.385	0.423	0.654	0.615	0.500

Additionally, we find that the ER_*mu*_ for the comparison of *H*_1_ with *H*_*u*_ is .500, indicating that approximately half of the individual Bayes factors expresses support for ${H_{1}^{i}}$, while the other half expresses support for ${H_{u}^{i}}$. Similarly, ER_2*u*_, ER_3*u*_ and ER_4*u*_ are .346, .615 and .423 indicating that for these hypotheses, too, there is little homogeneity among the individual Bayes factors. Only SR_1*u*_ is rather close to .50, and consequently, it is not likely that the gP-BF_*mu*_ is affected by one or more influential cases having a (much) smaller BF than the majority. For the other hypotheses, there is indication that the strength of the gP-BF_*mu*_ is affected by skewness among the individual Bayes factors.

Based on the gP-BF_*mu*_, ER_*mu*_, and SR_*mu*_, we can draw the following conclusions. Firstly, using the gP-BF_*mu*_ no hypothesis could be selected as the best hypothesis from the set. The SR_*mu*_s indicate that for all hypotheses but ${H_{1}^{i}}$ imbalance among individual Bayes factors was present. Furthermore, the relatively low ER_*mu*_s indicate that it is unlikely that the individuals come from a homogeneous population with respect to any of the specified hypotheses. Finally, none of the hypotheses appears to be a good description of the ordering of the individual success probabilities. Thus, based on these findings it seems unlikely the P-population is homogeneous with respect to the WP-population hypotheses that were considered.

A within-person experiment, such as conducted by Zedelius et al., (2011), is quite common in social and neuro-psychological research. The theory and hypotheses for these experiments are often at the WP-population level. Examples are Moreland and Zajonc (1982), who wonder “...*whether mere exposure to other people [...] is a sufficient condition for the enhancement of their perceived similarity to ourselves.*” (p. 397) and Klimecki et al., (2016), who hypothesize that “... *altruistic motivation is elicited by empathy felt for a person in need.*” (p. 1). Zedelius et al., (2011) write that “...*rewards cause people to invest more effort in a task*...”, “...*the intriguing hypothesis that [...] reflective thoughts hinder ongoing performance*...”(p. 355) and “...*participants performed significantly better*...” (p. 356). These fragments contain theory or expectations regarding the behavior of individual people.

Although WP-population hypotheses are formulated, the analyses are usually executed at the P-population level. In the original Zedelius et al., (2011) paper, the data were analyzed by means of a repeated measures ANOVA, which tests differences in the P-population means. The conclusions obtained from this analysis imply that *H*_2_ holds at the P-population level. Often the, usually implicit, assumption is that if a hypothesis holds at the P-population level, it holds for all individuals. The current analysis shows that although *H*_2_ is a reasonable hypothesis at the P-population level, it appears not to be the single best hypothesis under consideration and is not a good hypothesis for all individuals. The assumption that an average conclusion holds for all individuals is in this case violated. It is important that psychological researchers are aware of the fact that conclusions at the P-population level cannot be transferred to the individual level without testing this. Within-person experiments offer rich data that allow for the evaluation of individual hypotheses, through which the assumption that a hypothesis holds for everyone can be tested. This paper introduces an approach with which this can be done.

## Determining the sample size and number of replications for a study

Say a researcher has a research question that he wants to test by means of an experiment. This research question defines which and how many conditions *J* should be considered and results in one or multiple hypotheses of interest. The researcher is then left with two choices regarding the experiment, namely, the number of replications *R* used in each trial and the sample size *P*. This section will describe a method to choose *R* and *P*.

In the previous section, a method to evaluate a set of individual Bayes factors has been introduced in the form of three measures: gP-BF_*mu*_, ER_*mu*_ and SR_*mu*_. It is important to investigate the properties of these measures as a function of sample size and the number of replications. In other words, if indeed all individuals are homogeneous with respect to an individual informative hypothesis, which are the sample size and number of replications required for gP-BF_*mu*_, ER_*mu*_ and SR_*mu*_ to succeed in detecting this and, analogously, if individuals are not homogeneous, can this be derived from these measures?

Through a sensitivity analysis it can be determined for which sample size and number of replications the gP-BF_*mu*_ can be expected to prefer the hypothesis that is in agreement with the true P-population, the ER_*mu*_ is sufficiently high and SR_*mu*_ is close to .5. The choice for what values the gP-BF_*mu*_, ER_*mu*_ and SR_*mu*_ behave as desired is subjective. In line with our reasoning for the interpretation of individual Bayes factors as described on page 14, the choice for when the strength of support in gP-BF is sufficient to prefer one hypothesis over another is subjective and no guidelines are provided. Additionally, we will consider .9 to be sufficiently high for the ER_*mu*_, that is, a maximum 10*%* of individual Bayes factors prefers a different hypothesis than the majority, and a .1 margin around .5 to be reasonable for the SR_*mu*_, that is, the proportion of individual Bayes factors expressing stronger support than gP-BF_*mu*_ is between .4 and .6.

Using R version 3.3.1 (R Core Team, 2013), software has been developed with which such a study design analysis can be executed.^1^ While discussing the options of this program, we focus on the evaluation of , in order to arrive at an appropriate study design to determine whether ${H_{m}^{i}}$ holds for everyone in the P-population. The program can analogously be used for Study design analyses for gP-$BF_{mm^{\prime }}$ or gP-*B* *F*_*mu*_. The required input and the algorithm used are illustrated using Zedelius et al., (2011), as it could have been conducted before starting the data collection.

The R program requires as input the number of conditions *J* and hypotheses that a researcher wants to investigate. Additionally, the numbers of replications *R* and the sample sizes *P* that a researcher is willing to consider should be specified. Using this input, the following steps are executed:

- For each hypothesis of interest ${H_{m}^{i}}$, three *P*-populations are specified, one where ${H_{m}^{i}}$ is true for all WP-populations, one where is true for all WP-populations and a mixture of these two populations. In the next section these P-populations are specified in more detail for the example from Zedelius et al., (2011).
- For each P-population, the program generates 10,000 WP-populations, that is, parameter vectors ***π*** ^*i*^ of size *J*.
- For each *R* specified by the user, **x** ^*i*^ is sampled from ***π*** ^*i*^.
- For each **x** ^*i*^, is computed.

This results in 10,000 individual Bayes factors for each combination P-population and *R*. For computational reasons, this set will be used as a surrogate for the true infinite P-population. For each surrogate P-population then the following steps are followed:

- For each sample size *P* and number of replications *R*, 1000 sets of individual Bayes factors are sampled with replacement from the surrogate P-population.
- For each set, the gP-BF_*mu*_, ER_*mu*_ and SR_*mu*_ are computed, resulting in 1000 values of each measure for every sample size *P* and number of replications *R*.
- From these 1000 values of gP-BF_*mu*_, ER_*mu*_ and SR_*mu*_ the 2.5, 50 and 97.5 percentiles are obtained. The 50 percentile, the median, is used to summarize what values can be expected for each of these measures. The desired values of these expectations are, as described above subjectively defined, for the gP-BF_*mu*_, above .9 for the ER_*mu*_ and within a .1 margin from .5 for the SR. The 2.5 and 97.5 percentiles indicate the range in which 95*%* of the sampled gP-BF_*mu*_, ER_*mu*_ and SR_*mu*_ lay. If this range is very wide and includes non-reasonable values the combination of *R* and *P* might not be appropriate even when the expected value is of a desired level. In the next section we will illustrate how this information can be used to determine the *R* and *P* required to execute a study.

### Illustration

This section describes a sensitivity analysis for the determination of the number of replications *R* and sample size *P*, where the setup of Zedelius et al., (2011) will be used as starting point. Of course, such an analysis should be executed prior to the data collection, which was already done by Zedelius et al., (2011). However, for the illustration we will do the analysis as if no data has been collected yet. This will provide us with the knowledge whether the eventually chosen *R* and *P* were sufficient according to the sensitivity analysis. The first step of the sensitivity analysis described in the previous section requires a research question leading to the number of conditions *J* and a set of hypotheses representing the researchers’ expectations. The research question of Zedelius et al. rendered three hypotheses, Eqs. 9–11, about the ordering of success probabilities in the *J* = 8 experimental conditions. For this illustration, only ${H_{1}^{i}}$ as in Eq. 2 is considered. This results in the following parameters for the sensitivity analysis:

- *Number of conditions.* Zedelius et al., (2011) considered 8 different conditions, so *J* = 8.
- *Hypothesis.* The hypothesis that will be considered for this illustration is ${H_{1}^{i}}$. From this hypothesis, three relevant P-populations are derived.
  - *P-population 1.* In this P-population all individuals adhere to ${H_{1}^{i}}$. Using this population the median values of the gP-BF_*mu*_, ER_*mu*_ and SR_*mu*_ can be determined if ${H_{m}^{i}}$ holds for everyone. To compute these median values, the individual parameters ***π*** ^*i*^ are repeatedly sampled from the prior distribution under ${H_{1}^{i}}$:
    28 $$ h(\boldsymbol{\pi}^{i}|{H_{1}^{i}}) \propto h(\boldsymbol{\pi}^{i}|{H_{u}^{i}})I_{\boldsymbol{\pi}^{i} \in {H_{1}^{i}}},  $$
    where $I_{\boldsymbol {\pi }^{i} \in {H_{1}^{i}}} = 1$ if ***π*** ^*i*^ is in agreement with ${H_{1}^{i}}$ and 0 otherwise.
  - *P-population 2.* In this P-population all individuals adhere to . Using this population, the expected values of the gP-BF_*mu*_, ER_*mu*_ and SR_*mu*_ can be determined if are sampled from the prior distribution under , that is: 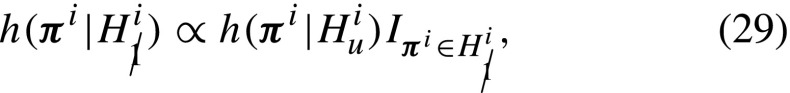

    where if ***π*** ^*i*^ is in agreement with and 0 otherwise.
  - *P-population 3.* For the third P-population, a mixture of P-population 1 and 2 is considered. Using this population, the expected values of the gP-BF, ER and SR can be determined if ${H_{m}^{i}}$ holds for a proportion *𝜃* of individuals in the P-populations, and holds for a proportion 1 − *𝜃* of individuals. The individual parameters ***π*** ^*i*^ are sampled from Eq. 26 if *u* ^*i*^, sampled from *U*(0,1) is smaller than or equal to the specified proportion *𝜃*, and sampled from Eq. 29 if *u* ^*i*^ is larger than *𝜃*: 
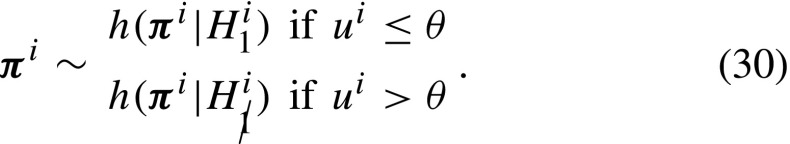

  The proportion *𝜃* is set to .5, thus half of all individuals adheres to ${H_{1}^{i}}$ and the other half adheres to .

Next, the sample sizes *P* and number of replications *R* that the researchers want to consider should be chosen. Based on the choices made by Zedelius et al., (2011), the following values for *P* and *R* are considered for the sensitivity analysis:

- *Number of replications.* Zedelius et al.,^2011^ used seven replications in their experiment. Additionally, it would be interesting whether more replications would result in better performance, therefore *R* = 7,14,21 are considered.
- *Number of individuals.* Zedelius et al.,^2011^ used 26 participants in their experiment. In order to mimic an a priori sensitivity analysis, the sample sizes*P* = 5,7,10,15,20,25,30,40,50 are considered.

### Results

**Fig. 2:**
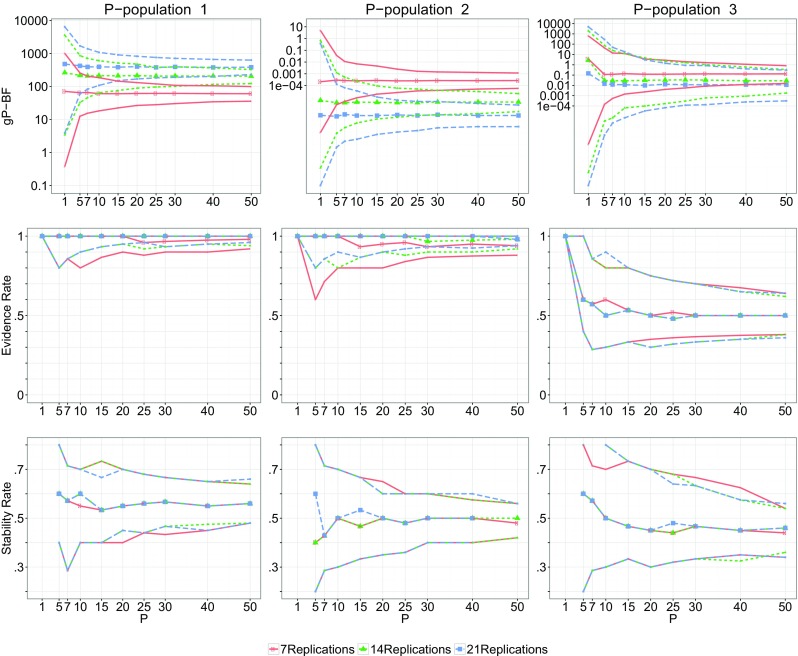
, and for the three generated true P-populations for *J* = 8. P-population 1 is described in Eq. 26, P-population 2 in Eq. 29, and P-population 3 in Eq. 30. Both the median and 95*%* interval are shown in the figures

Figure 2 shows the results of the sensitivity analysis for the determination of sample size *P* and number of replications *R*. The results are presented for each of the three simulated P-populations described in the previous section. The first column of the figure shows the performance of the gP-BF_*mu*_, ER_*mu*_ and SR_*mu*_ if ${H_{1}^{i}}$ is true for all individuals (P-population 1). As can be seen in the top left figure, already for small sample sizes the gP-BF_*mu*_ expresses strong support for *H*_1_: the lower 2.5 percentile of the gP-BF_*mu*_ is larger then 10 for *R* > 7 and *P* > 5. The lower 2.5th percentile of the ER_*mu*_ only stabilizes above .9 for *R* = 7 and *P* > 30 and for *R* = 14,21, this is already achieved for *P* > 10. Stated otherwise, if ${H_{1}^{i}}$ holds for all individuals, for samples larger than 30 it is likely that less then 10 per cent of individual Bayes factors express support for . Finally, the bottom panel shows that the SR_*mu*_ stabilizes around .55, reflecting that it is reasonable to expect slightly more than half of the individual Bayes factors to express stronger support than gP-BF_*mu*_. This implies that the gP-BF_*mu*_ is, on average, slightly more influenced by the ‘weaker’ and contradicting individual Bayes factors. The 2.5 and 97.5 percentiles are within a margin of .1 from the median gP-BF for *P* > 25. Furthermore, we see that from around *P* = 25 the median and 2.5 and 97.5 percentiles stabilize. Thus, if ${H_{1}^{i}}$ is true for all individuals, with sample size *P* around 25 − 30 and *R* = 7, the gP-BF_*mu*_ and ER_*mu*_ perform as desired: the gP-BF_*mu*_ shows strong evidence for the true hypothesis, the ER_*mu*_ is high and the SR_*mu*_ is around .5.

In the middle column of figures in Fig. 2 is true for all individuals. For *P* > 10 and *R* > 7, the gP-BF is smaller than .01, indicating at least ten times more support for than for ${H_{1}^{i}}$. As *R* increases, so does the median support found in the data. The lower 2.5 percentile of the ER is above .9 for *P* > 30 and *R* = 14,21 and close to .9 for *R* = 7. The median SR is almost exactly .5 for all *R* for *P* > 20, and the 2.5 and 97.5 percentiles are within .1 of the median for *P* > 30. Thus, for sample sizes of 30 and larger, the gP-BF_*mu*_, ER_*mu*_ and SR_*mu*_ behave as desired for *R* = 7 and even better for *R* = 14,21.

Finally, Population 3, depicted in the right column in Fig. 2 was chosen to be a mixture of the first two populations. Here it can be seen that if ${H_{1}^{i}}$ holds for 50*%* of the individuals in the population, generally, is preferred over ${H_{1}^{i}}$, although with less strength than when Population 2 was the true population. Note that this happens because it is more likely that a person coming from $h(\boldsymbol {\pi }^{i}|{H_{1}^{i}})$ provides evidence in agreement with than vice versa. For example, if *H*_1_ is true but if the ordering in the data is off by one order constraint, we are likely to prefer . However, if one of the orderings that comprises is true, a ‘mistake’ in one or more of the order constraints in the data does not necessarily lead to a preference for *H*_1_, but might point to one of the other orderings under . The complexity of *H*_1_ is 2.48 × 10^− 5^ and the complexity of $H_{\tilde {A}\text {\u {g}}ancel{1}}$ is 1 − 2.48 × 10^− 5^ ≈ 1. Thus, even though *𝜃* = .5, is preferred because it has a higher complexity. The is of use here, indicating that there are multiple populations and stabilizing around .5 for *P* > 30. Although the median support found in the might indicate a preference for over ${H_{1}^{i}}$, the indicates inconsistency among individual Bayes factors. Finally, the median for this population is slightly below .5, and the 2.5 and 97.5 percentiles are further than .1 from this median until *P* is around 40, for *R* = 14,21 or 50 for *R* = 7. Thus, if neither of the two hypotheses hold for everyone, this is reflected in the for every *P* and *R* that seemed reasonable if ${H_{1}^{i}}$ or were true for everyone.

Zedelius et al., (2011) eventually used 26 participants in their study and seven replications. This is slightly lower than the suggested 30 based on the sensitivity analysis. Consulting the figures, it seems that, if ${H_{1}^{i}}$ is true and *P* = 26 and *R* = 7, is expected to be between 30 and 100, the is expected to be above .9 and the between .43 and .67. On the other hand, if is true for all individuals, the can be expected between 1000 and 10,000 in support of , with the similarly above .9 and the between .35 and .6. Consulting the results in Table 4, we find that , ER_*mu*_ = .500 and SR_*mu*_ = .436. These results do not seem in line with either Population 1 or 2, but consulting the right column figures in Fig. 2, they do seem in line with the mixture population. Of course, this is no evidence that indeed this mixture population with *𝜃* = .5 is the most likely true P-population. However, it does indicate that even though the shows some support for relative to ${H_{1}^{i}}$, it is not likely that holds for everyone in the P-population.

## Discussion

After formulating within-person (WP) hypotheses, individual Bayes factors can be computed with which the support for a particular hypothesis can be derived for each person, or the best from a set of informative hypotheses can be selected. A method has been proposed to combine the individual Bayes factors of some, in order to draw conclusions for all - by answering the question whether an individual hypothesis holds for all persons in the population - and for one by determining the average support for ${H_{m}^{i}}$ relative to $H_{m^{\prime }}^{i}$ which describes what could be expected for a next individual. The geometric average of *P* individual Bayes factors (gP-BF) describes the average support for one hypothesis relative to another. It describes what individual Bayes factor could be expected for a next person. Together with the evidence rate and stability rate, the gP-BF can be used to assess whether one hypothesis is more supported than another for all individuals in a population. By means of a sensitivity analysis for a set of hypotheses, it can be determined for what sample size *P* and number of replications *R* in an experiment these measures behave desirable.

An R Shiny application has been developed with which a sensitivity analysis can be executed prior to data collection. By specifying hypotheses of interest, the behavior of gP-BF, ER and SR can be evaluated for various combinations of *R* and *P*. This allows researchers to collect the appropriate data for their question of interest. Besides an own sensitivity analysis, the data of the simulations used as examples in this paper can be accessed and viewed within the application. Furthermore, in the application data can be analyzed and the gP-BF, ER, and SR are computed. The application and manual can be accessed on https://github.com/fayetteklaassen/OneForAll.

## Appendix: Computation of fit and complexity through decomposition

In order to compute the Bayes factor that expresses the support in favor of *H*_*m*_:

31 $$ H_{m}: R_{m}\boldsymbol{\pi} > 0 $$

against the unconstrained hypothesis *H*_*u*_, the complexity and fit of *H*_*m*_ should be computed.^2^

Complexity and fit can be determined by taking samples from the unconstrained prior and posterior distribution respectively. A common approach is to take *Q* samples, and determine what proportion of the samples is in agreement with *H*_*m*_, such that

32 $$\begin{array}{@{}rcl@{}} f_{m} &=& {\int}_{\boldsymbol{\pi}\in H_{m}} g(\boldsymbol{\pi}|\mathbf{x}, H_{u})\delta \boldsymbol{\pi}\\ &\approx& \frac{1}{Q}\sum\limits_{q = 1}^{Q}I_{\boldsymbol{\pi}^{q}\in H_{m}} \end{array} $$

where ***π*** ^*q*^ is the *q* th sample from the unconstrained posterior and $I_{\boldsymbol {\pi }^{q}\in H_{m}} = 1$ if ***π*** ^*q*^ is in agreement with *H*_*m*_, and 0 otherwise. The complexity can be computed analogously, with the difference that samples are taken from the prior distribution.

If *H*_*m*_ concerns the ordering of 8 parameters, the complexity can be derived analytically and is 1/8! = 1/40, 320. Using *Q* = 100, 000 samples from the unconstrained prior only 2 or 3 samples of ***π*** are expected to adhere to the constraints under *H*_*m*_. This implies that the estimate of *f*_*m*_ is very unstable. To obtain stable estimates impossibly huge samples are needed. Similarly, the fit of a hypothesis with eight parameters might be too small to accurately approximate using 100,000 samples. One solution is to increase the number of samples which increases the computational time. Mulder, Hoijtink, and de Leeuw (2012) present another solution that makes use of a decomposition of the complexity and fit. This procedure determines decomposed fit and complexity for each constraint in a hypothesis. Equation 29 shows how the probability that all constraints hold, given *H*_*u*_ and the data **x**, can be rewritten as a product of decomposed probabilities:

33 $$\begin{array}{@{}rcl@{}} P(\mathbf{R}_{m}\boldsymbol{\pi} > 0|H_{u}, \mathbf{x}) &=& \prod\limits_{k = 1}^{K} P(\mathbf{R}_{m}^{(k)}\boldsymbol{\pi}>0|H_{u}, \mathbf{x}, \mathbf{R}_{m}^{(1:k-1)})\\ &=& \prod\limits_{k = 1}^{K} f_{m}^{(k)}\\ &\approx& \prod\limits_{k = 1}^{K}\frac{1}{Q}\sum\limits_{q = 1}^{Q} I_{\mathbf{R}_{m}^{(k)}\boldsymbol{\pi}^{q}>0}, \end{array} $$

where *K* is the number of constraints in hypothesis *m*, $\mathbf {R}_{m}^{(k)}$ is the *k* ^th^ row of **R**_*m*_, $\mathbf {R}_{m}^{(1:k-1)}$ are the first *k* − 1 rows of **R**_*m*_, $f_{m}^{(k)}$ is the decomposed fit for the *k* ^th^ constraint, the indicator function $I_{\mathbf {R}_{m}^{(k)}\boldsymbol {\pi }^{q}>0} = 1$ if $\mathbf {R}_{m}^{(k)}\boldsymbol {\pi }^{q}>0$ and 0 otherwise and ***π*** ^*q*^ is sampled from $g(\boldsymbol {\pi }|H_{u}, \mathbf {x}, \mathbf {R}_{m}^{(1:k-1)})$.

Since each $f_{m}^{(k)}$ is only defined by one constraint, it is never a small value and can be estimated with relatively few samples. The R Shiny application *OneForAll* belonging to this paper uses *Q* = 10, 000. By multiplying the decomposed fit components similar to Eq. 29 the total fit can be obtained accurately.

The complexity can be derived analogously:

34 $$\begin{array}{@{}rcl@{}} P(\mathbf{R}_{m}\boldsymbol{\pi} > 0|H_{u}) &= &\prod\limits_{k = 1}^{K} P(\mathbf{R}_{m}^{(k)}\boldsymbol{\pi}>0|H_{u}, \mathbf{R}_{m}^{(1:k-1)})\\ &=& \prod\limits_{k = 1}^{K} c_{m}^{(k)}\\ &\approx& \prod\limits_{k = 1}^{K}\frac{1}{Q}\sum\limits_{q = 1}^{Q} I_{\mathbf{R}_{m}^{(k)}\boldsymbol{\pi}^{q}>0}, \end{array} $$

where $c_{m}^{(k)}$ is the decomposed complexity conditional for the *k* ^th^ constraint and ***π*** ^*q*^ is sampled from $h(\boldsymbol {\pi }|H_{u}, \mathbf {R}_{m}^{(1:k-1)})$.

## Footnotes

1 The software with accompanying manual can be downloaded on https://github.com/fayetteklaassen/OneForAll, or be obtained by contacting the first author at klaassen.fayette@gmail.com. For assistance with or questions about the software, please also contact the first author.
